# Does the apex optimization line matter for single‐channel vaginal cylinder brachytherapy planning?

**DOI:** 10.1002/acm2.12351

**Published:** 2018-05-16

**Authors:** Yusung Kim, Katherine Cabel, Wenqing Sun

**Affiliations:** ^1^ Department of Radiation Oncology Carver College of Medicine Iowa, City IA USA; ^2^ Department of Biomedical Engineering College of Engineering The University of Iowa Iowa, City IA USA

**Keywords:** cervical cancer, high‐dose‐rate brachytherapy, high‐dose‐rate brachytherapy treatment planning, optimization, vaginal cylinder planning

## Abstract

The objective of this study is to test the impact of the use of the apex optimization line for new vaginal cylinder (VC) applicators. New single channel VC applicators (Varian) that have different top thicknesses but the same diameters as the old VC applicators (2.0 cm diameter, 2.3, 2.6, 3.0, and 3.5 cm) were compared using phantom studies. Old VC applicator plans without the apex optimization line were also compared to the plans with an apex optimization line. The apex doses were monitored at 5 mm depth doses (eight points) where a prescription dose (Rx) of 6 Gy was prescribed. VC surface doses (eight points) were also analyzed. The new VC applicator plans without apex optimization line presented significantly lower 5‐mm depth doses over the Rx (on average −31 ± 7%, *P* < 0.00001) due to thicker VC tops (3.4 ± 1.1 mm thicker with the range of 1.2–4.4 mm) than the old VC applicators. Old VC applicator plans also showed a statistically significant reduction (*P* < 0.00001) due to the Ir‐192 source anisotropic effect at the apex region, but the percent reduction over the Rx was only −7 ± 9%. However, by adding the apex optimization line to the new VC applicator plans, the plans improved 5‐mm depth doses (−7 ± 9% over Rx) that were not statistically different from old VC applicator plans (*P* = 0.923), along with apex VC surface doses (−22 ± 10% over old VC vs −46 ± 7% without using apex optimization line). The use of the apex optimization line is important in order to avoid significant additional cold doses (−24 ± 2%) at the prescription depth (5 mm) of the apex, specifically for the new VC applicators that have thicker tops. A template‐based vaginal cylinder planning reduced the intra‐ and inter‐planner variations of manual generation of apex optimization line, along with treatment time.

## 1 INTRODUCTION

Endometrial cancer is the most common gynecologic cancer in the United States and worldwide. In 2015, an estimated 54,870 women were diagnosed with endometrial cancer resulting in an estimated 12,900 deaths in the United States alone.^1^ After hysterectomy and bilateral salpingo‐oophorectomy, the vagina is the most frequent site of recurrence for endometrial cancer.^2^ Post‐operative vaginal brachytherapy (BT) without external beam radiotherapy (EBRT) was found to be as effective as EBRT by ensuring vaginal control with few gastrointestinal toxic effects when treating high to intermediate risk endometrial cancer.^2^ The estimated 5‐year recurrence rates after treatment with either vaginal BT or EBRT^2^, ^3^ were similar (1.8% and 1.6%) and showed no significant difference in 5‐year locoregional recurrence and distant metastases rates. The American Brachytherapy Society (ABS) survey reported that vaginal BT is a common recommendation for post‐operative adjuvant therapy for endometrial cancer.^3^ Following surgery, the vaginal canal for most patients is roughly cylindrical, and the ABS recommends a properly sized, single‐channel vaginal cylinder applicator (VC) for BT treatment.^4^ The VC is the most common applicator used for high‐dose‐rate (HDR) BT^3^ and is ideal for patients with a narrow vagina.^4^ The region including the vaginal cuff accounts for about 75% of recurrences in endometrial cancer patients.^2^, ^4^, ^5^ It is important to generate a radiation dose distribution that best conforms to the vaginal cuff region through optimization during treatment planning. The most recent ABS recommendations (released in 2012), define optimization as the manipulation of the HDR BT dwell positions, dwell times, or both.^4^ The ABS recommends using an optimization line at the upper apex or vaginal cuff as well as the lateral areas in order to avoid unacceptably high doses to the vaginal apex and any overlying portion of the small bowel.^4^, ^5^ Delivering a radiation dose to the vaginal cuff that receives the prescription dose (Rx) as much as possible is desirable. At least its considerably cold doses should be avoided during planning procedure as the risk of recurrence at the vaginal cuff is greater than 70%.^2^, ^4^, ^5^ Our institution had not previously applied the practice of using an apex optimization line, and consequently significant under‐doses to the apex region were observed by a physician on a VC HDR plan. This study demonstrates how a new VC applicator, from the same vendor with the same diameter, can cause significant under doses at the vaginal cuff where the risk of recurrence is greatest. Additionally, we introduce a commissioning process using template‐based VC planning in order to avoid errors incurred during the manual generation of the apex optimization lines.

## 2 MATERIALS AND METHODS

### 2.A New and previously used VC applicators

**Figure 1:**
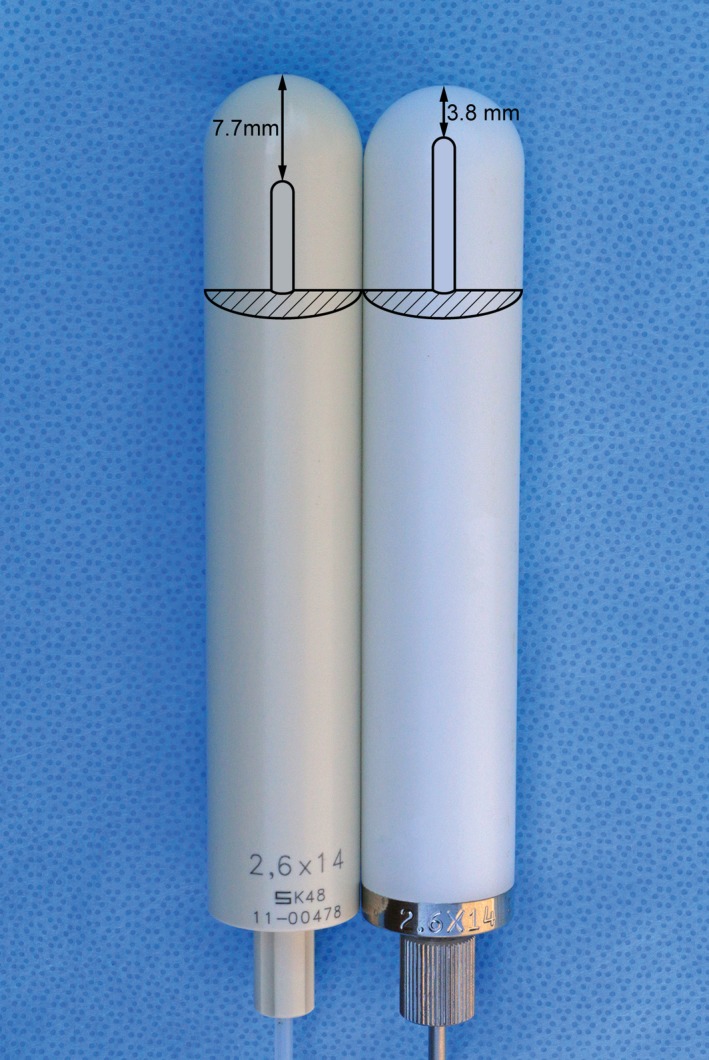
Comparison of new (LEFT) and discontinued, old (RIGHT) vaginal cylinder applicators, and their inside diagrams showing varying top thicknesses.

This planning study compares vaginal cuff doses using plans generated from both a new and discontinued VC applicator. All new and discontinued VC applicators used in our clinic are single channel applicators from the same vendor (Varian Medical Systems, Inc., Palo Alto, CA). New VC applicators have the same diameter as the discontinued applicators but have a different top thickness, (see summary in Table 1). The top thickness values of both VC applicators in Table 1 are the nominal values provided by the vendor. The 2.6‐cm diameter applicator is demonstrated in Fig. 1. For illustration purposes, the top thickness dimensions were drawn using a scale of 1:2. New VC applicators are designed with the new guide tubes directly connected to the tandem of the VC applicator, while old catheters were inserted inside the tandem of the VC applicators. These new guide tubes and VC applicator are the so‐called “ClickFit” VC applicators. They are transparent so that clinicians may visually check whether a source wire is in position or out during an emergency situation. The diameters of the five old and new VC applicators (2.0, 2.3, 2.6, 3.0, and 3.5 cm) were compared.

**Table 1:** The top thickness of old and new VC applicators

		Single‐channel vaginal cylinder diameter (cm)				
		2.0	2.3	2.6	3.0	3.5
Top thickness (cm)	Old VC	1	2.4	3.8	5.8	9.9
	New VC	5.4	6.5	7.7	9.2	11.1
	Diff^a^	4.4	4.1	3.9	3.4	1.2

a Difference = New VC − Old VC.

### 2.B VC HDR program and phantom planning study design

An institutional VC HDR prescription typically comprises a total of 18 Gy in three fractions of 6 Gy per fraction (without EBRT); prescribed to a depth of 5 mm from the VC applicator surface. Active dwell‐position lengths are typically 5 cm. All cases in this phantom study have a 5‐cm active dwell‐position length. As a matter of routine in our VC HDR workflow, a single treatment plan is generated after taking a computerized tomography (CT) scan after a VC insertion. This single plan is used for three distinct VC HDR deliveries by scaling the dwell times of each treatment, relative to the anterior–posterior (AP) x‐ray image taken on each treatment day and the simulation day CT. During the treatment planning procedure, the two lateral 5‐mm depth lines are used to optimize dwell times and corresponding isodose lines using BrachyVision v10.0 (Varian Medical Systems, Inc.). The resulting isodose lines are specific to the VC size and not to the patient's anatomy. Bladder and rectal doses, which are specific to each patient's anatomy, are checked on each plan. A half‐circle line of bladder points is drawn on the foley on the sagittal view of the CT image. Two rectal lines are also drawn: (a) rectal outer surface line, and (b) a 5‐mm depth line from the rectal outer surface. Their maximum doses are then confirmed to be less than 80% of the Rx (6 Gy). When the 7 Gy Rx exceeded each OAR dose limit, especially during EBRT treatment, the Rx was lowered to diminish the risk of toxicity.

The apex optimization line was retrospectively added at a 5‐mm depth from the VC surface for the new VC applicator plans. This apex optimization line consists of eight points of equal distance. Their average dose was compared to the Rx when the apex optimization lines were included in the optimization process. The 5‐mm depth dose of the discontinued applicators was also compared to the Rx without using the apex optimization line. The VC surface doses of the eight points, along with the apex 5‐mm depth doses were also monitored.

As the apex 5‐mm depth doses are dependent on the VC size and not on patient anatomy, this study was performed as a phantom planning study.

### 2.C Commissioning a template‐based VC plan

Because the apex optimization line requires the manual generation of eight points at a 5‐mm depth in the apex region, inter‐ and even intra‐user variations are inevitable. To avoid any user‐induced variation, a template‐based VC plan was commissioned using CT or x‐ray images as the primary treatment planning dataset. Backup x‐ray image‐based VC planning templates were generated in case of CT scanner malfunction. Each new VC applicator was originally designed to be used with a flexible plastic probe with a tip thickness of 1.7 mm. However, in order to clamp a VC probe to an air hover based HDR patient transfer system (Zephyr, Diacor Inc., Salt Lake City, Utah), a stainless steel probe with a tip thickness of 0.3 mm had to be substituted for the plastic applicator. The nominal value of the stainless steel top thickness (0.3 mm) was independently validated using x‐ray images due to the institutional limitation of a minimum 0.6‐mm CT slice thickness. Due to a top thickness difference of 1.4 mm between the old and the new VC applicators, coronal and sagittal VC surface reference lines were added to the treatment plan when a template was generated for each size of a new VC applicator. A total of five templates were created by generating VC treatment plans for each VC size using CT image datasets. These datasets comprised two lateral 5‐mm depth optimization lines one right and one left, an apex 5‐mm depth optimization line, as well as coronal and sagittal VC surface reference lines. Each VC template plan had a 5‐cm active length with a prescription of 6 Gy, which is our institutional standard of care.

## 3 RESULTS AND DISCUSSION

**Figure 2:**
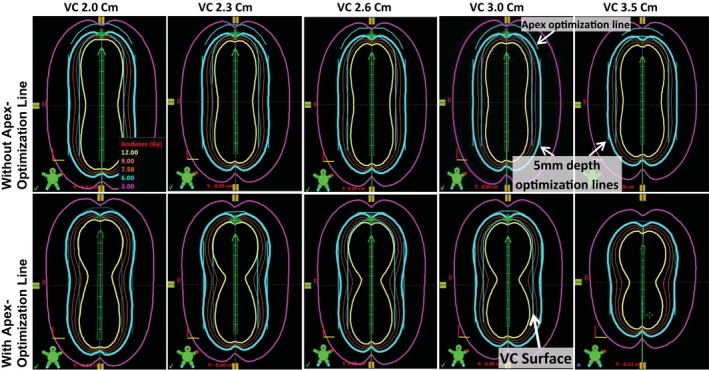
Comparison of isodose lines with and without apex optimization of a new vaginal cylinder applicator (VC) for five different VC sizes.

**Figure 3:**
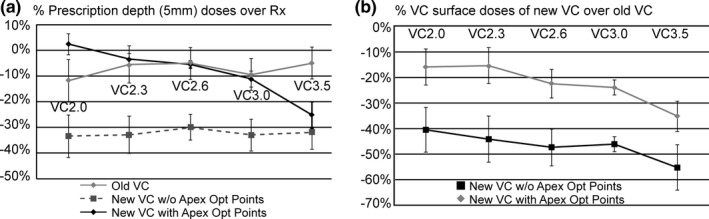
The percent prescription depth (5 mm) doses comparison of the old VC, the new vaginal cylinder applicator(VC) without apex optimization line and with the points presents significant under doses when the new VC is used without using apex optimization line (LEFT). The %VC surface doses of the new VC without using apex optimization line also presented significantly lower than the new VC with the apex optimizationline when compared to those of the old VC (RIGHT).

New single‐channel VC applicators (GM11004760, Varian Medical Systems) that have a thicker top but the same diameter as the old VC applicators (2.0 cm diameter, 2.3, 2.6, 3.0, and 3.5 cm) were compared using phantom studies. The new VC applicator plans created without using the apex optimization line presented significantly lower prescription depth (5 mm) doses than the Rx (on average −31 ± 7%, *P* < 0.00001) due to their thicker tops (3.4 ± 1.1 mm thicker with the range of 1.2 to 4.4 mm) (see Figs. 2 and 3). The old VC applicator plans also showed a statistically significant dose reduction (*P* < 0.00001) due to the Ir‐192 source anisotropic effect at the apex region when an apex optimization line was not used, but the percentage reduction over prescription was only −7 ± 9%. Even though the ABS recommendations^4^, ^5^ cautions against unacceptably high doses to the vaginal apex when an apex optimization line is not used, we found significantly lower doses, on average — 7%, to the apex region when an old VC applicator was used without an apex optimization line. By adding the apex optimization line to the new VC applicators, the plans improved the 5‐mm depth doses (−7 ± 9% over Rx) and apex VC surface doses (−22 ± 10% over old VC vs −46 ± 7% without using apex optimization line) that were not statistically different from the old VC plans (*P* = 0.923) (see Figs. 2 and 3). The use of the apex optimization line is important in order to avoid significant additional cold doses (−24 ± 2%) at the apex prescription depth of 5 mm, specifically for the new VC applicators that have the thicker tops. The warnings in relation to the unexpected dosimetric changes found in this study can be applied to the commissioning process whenever existing applicators are replaced with new products. For example, a relatively newer single‐channel VC applicator (Universal Stump Applicator, GM11011160, Varian Medical Systems) is now available through the vendor. It should be conscientiously commissioned, with great care applied to its validation on the 3D‐modeled applicator library and attention paid to its physical dimensions and how they vary from legacy devices.

A total of five VC template plans for each VC size that vastly reduce the planning time were successfully generated. The templates allow the clinician to simply align the pre‐generated VC plans to the correct anatomical position of the cylinder on the CT image then define the three references lines (bladder foley, rectal surface line, and 5‐mm depth rectal line). These are the only patient‐specific anatomical reference lines. VC planning is specific to patients in order to monitor organs‐at‐risk (OARs) doses. We chose not to use the VC applicator library (Solid Applicator, Varian Medical Systems, Inc.) available in the treatment planning system (BrachyVision v10.0, Varian Medical Systems, Inc.), since we did not use the plastic‐based flexible probe which differs by 1.4 mm in top thickness from the stainless steel probe. Clinicians must validate the dimensions of the 3D‐modeled applicator library before clinical use, especially when a new version of an applicator is released without a corresponding update to the applicator library. As an example of the type of errors found on 3D‐modeled applicator libraries, Kim et al.^6^ found that the right ovoids of a titanium Fletcher‐Suit‐Delclos‐style tandem‐and‐ovoids set incorrectly matched with the left ovoids in the provided 3D‐modeled applicator library. The use of a stainless steel probe, instead of its original plastic‐based flexible probe, can cause dosimetric changes due to its metal‐based material. Nonetheless, AAPM TG43 dose calculations^7^, ^8^, ^9^ were clinically performed, instead of the use of a model‐based heterogeneous dose calculation due to the minimal expected dosimetric impact of the effect of a metal applicator. For instance, Hyer et al.^10^ reported that the dosimetric impact of titanium on tandem‐and‐ovoids HDR brachytherapy plans was less than 3% when compared to plans generated when AAPM TG43 dose calculations were used.

The rationale for the use of apex optimization lines was the potential of unacceptably high doses at the vaginal apex and any part of the overlying small bowel when using an older VC applicator with a thinner top.^4^, ^5^, ^11^ When implementing the VC HDR BT program at our institution using the now‐discontinued VC applicators, staff physicians reviewing the resulting isodose lines were most focused on checking the Rx coverage of the vaginal cuff region. This was because VC HDR BT patients did not receive an additional EBRT boost. ABS guidelines^4^ suggest that the use of HDR optimization points on only the lateral 5 mm depth from the VC surface without having an apex optimization line would produce unacceptably high doses at the vaginal apex and any overlying area of the small bowel. That is due to the fact that older VC applicators have a thinner top than the VC applicators replacing them. Our findings presented new VC applicators with thicker tops requiring an additional apex optimization line during the isodose line generation process. For clinics already using the new VC applicators but not yet using an apex optimization line, there are systematic, unintended under doses (on average, −24 ± 2%) depending on the VC sizes, although the diameters are identical. In order to deliver the prescription dose to the apex region where the risk of recurrence is greatest, the use of the apex optimization line is recommended regardless of which version of single‐channel VC applicator (i.e., regardless of top thickness of the VC applicator) is used, in accordance with ABS guidelines.^4^ The clinical outcomes for VC HDR BT have shown favorable results with a 1.8% 5‐year vaginal recurrence rate, 2.1% locoregional recurrence (pelvic, vaginal, or both), and a 90.4% 3‐year overall survival rate.^2^, ^12^ Kella‐Sleczka et al. retrospectively analyzed adjuvant HDR VC BT patients with stage I–II endometrial cancer and found a 4‐year overall survival rate of 97%.^13^ The 2‐year relapse rate was 2% and no toxicities above grade 1 were observed.^13^ Unlike EBRT, VC brachytherapy has the potential for a better quality of life after radiation therapy due to fewer gastro‐intestinal side effects.^2^ VC HDR BT is often preferred as an adjuvant therapy following surgery in patients with an intermediate to high risk of endometrial cancer.^2^, ^12^, ^14^, ^15^, ^16^, ^17^, ^18^

Single‐channel VC applicators do have limitations such as the difficulty of sculpting dose away from the OARs. Due to its radially symmetrical dose distribution, the single‐channel method offers fewer possibilities to shape the isodose lines. To generate conformal isodose lines, a multi‐channel applicator has been developed^19^ that, unlike its single‐channel counterpart, is able to significantly reduce dose to OARs while optimizing target coverage.^19^ A study using a 13 channel Capri applicator (Varian Medical Systems, Inc.), showed similar target coverage to the VC applicator.^19^ However, the Capri applicator significantly decreased dosage to OARs (*P* < 0.00011) while optimizing target coverage.^19^ The additional channels at the periphery of the applicator may allow better dosimetry and reduce the unnecessary dosage to the bladder and rectum compared with a single‐channel applicator.^19^ For institutions using multi‐channel applicator‐based planning, it is still essential that a prescribed dosage is properly delivered to the apex region.

## 4 CONCLUSIONS

The use of apex optimization lines in treatment planning is important in order to avoid significant additional cold doses (−24 ± 2%) at the prescription depth (5 mm) of the vaginal apex, specifically for the new VC applicators that have thicker tops. A template‐based vaginal cylinder planning method reduced the intra‐ and inter‐planner variations inherent with the manual generation of the apex optimization line as well as reducing the treatment planning time.

## CONFLICT OF INTEREST

No conflict of interest.

## References

[1] The American Cancer Society . Cancer Facts & Figures. Atlanta: American Cancer Society; 2017.

[2] Nout RA , Smit VT , Putter H , et al. Vaginal brachytherapy versus pelvic external beam radiotherapy for patients with endometrial cancer of high‐intermediate risk (PORTEC‐2): an open‐label, non‐inferiority, randomised trial. Lancet. 2010;375:816–823. 20206777 10.1016/S0140-6736(09)62163-2

[3] Small W Jr. , Erickson B , Kwakwa F . American Brachytherapy Society survey regarding practice patterns of postoperative irradiation for endometrial cancer: current status of vaginal brachytherapy. Int J Rad Oncol Biol Phys. 2005;63:1502–1507. 10.1016/j.ijrobp.2005.04.038 16109462

[4] Small W Jr. , Beriwal S , Demanes DJ , et al. American Brachytherapy Society consensus guidelines for adjuvant vaginal cuff brachytherapy after hysterectomy. Brachytherapy. 2012;11:58–67. 22265439 10.1016/j.brachy.2011.08.005

[5] Nag S , Erickson B , Thomadsen B , et al. The American Brachytherapy Society recommendations for high‐dose‐rate brachytherapy for carcinoma of the cervix. Int J Radiat Oncol Biol Phys. 2000;48:201–211. 10924990 10.1016/s0360-3016(00)00497-1

[6] Kim Y , Modrick JM , Pennington EC , et al. Commissioning of a 3D image‐based treatment planning system for high‐dose‐rate brachytherapy of cervical cancer. J Appl Clin Med Phys. 2016;17:5818. 10.1120/jacmp.v17i2.5818 PMC5874852 27074463

[7] Nath R , Anderson LL , Luxton G , et al. Dosimetry of interstitial brachytherapy sources: recommendations of the AAPM Radiation Therapy Committee Task Group No. 43. American Association of Physicists in Medicine. Med Phys. 1995;22:209–234. 7565352 10.1118/1.597458

[8] Rivard MJ , Ballester F , Butler WM , et al. Supplement 2 for the 2004 update of the AAPM Task Group No. 43 Report: joint recommendations by the AAPM and GEC‐ESTRO. Med Phys. 2017;44:e297–e338. 28644913 10.1002/mp.12430

[9] Rivard MJ , Coursey BM , DeWerd LA , et al. Update of AAPM Task Group No. 43 Report: a revised AAPM protocol for brachytherapy dose calculations. Med Phys. 2004;31:633–674. 15070264 10.1118/1.1646040

[10] Hyer DE , Sheybani A , Jacobson GM , et al. The dosimetric impact of heterogeneity corrections in high‐dose‐rate (1)(9)(2)Ir brachytherapy for cervical cancer: investigation of both conventional Point‐A and volume‐optimized plans. Brachytherapy. 2012;11:515–520. 22386723 10.1016/j.brachy.2012.01.011

[11] Li Z , Liu C , Palta JR . Optimized dose distribution of a high dose rate vaginal cylinder. Int J Rad Oncol Biol Phys. 1998;41:239–244. 10.1016/s0360-3016(98)00014-5 9588940

[12] Nout RA , Putter H , Jurgenliemk‐Schulz IM , et al. Quality of life after pelvic radiotherapy or vaginal brachytherapy for endometrial cancer: first results of the randomized PORTEC‐2 trial. J Clin Oncol. 2009;27:3547–3556. 19546404 10.1200/JCO.2008.20.2424

[13] Kellas‐Sleczka S , Wojcieszek P , Bialas B . Adjuvant vaginal brachytherapy as a part of management in early endometrial cancer. J Contemp Brachytherapy. 2012;4:247–252. 23378855 10.5114/jcb.2012.32560 PMC3561608

[14] MacLeod C , Fowler A , Duval P , et al. High‐dose‐rate brachytherapy alone post‐hysterectomy for endometrial cancer. Int J Radiat Oncol Biol Phys. 1998;42:1033–1039. 9869226 10.1016/s0360-3016(98)00292-2

[15] Eltabbakh GH , Piver MS , Hempling RE , et al. Excellent long‐term survival and absence of vaginal recurrences in 332 patients with low‐risk stage I endometrial adenocarcinoma treated with hysterectomy and vaginal brachytherapy without formal staging lymph node sampling: report of a prospective trial. Int J Radiat Oncol Biol Phys. 1997;38:373–380. 9226326 10.1016/s0360-3016(97)00040-0

[16] Sorbe B , Straumits A , Karlsson L . Intravaginal high‐dose‐rate brachytherapy for stage I endometrial cancer: a randomized study of two dose‐per‐fraction levels. Int J Radiat Oncol Biol Phys. 2005;62:1385–1389. 16029797 10.1016/j.ijrobp.2004.12.079

[17] Chong I , Hoskin PJ . Vaginal vault brachytherapy as sole postoperative treatment for low‐risk endometrial cancer. Brachytherapy. 2008;7:195–199. 18358790 10.1016/j.brachy.2008.01.001

[18] Gadducci A , Greco C . The evolving role of adjuvant therapy in endometrial cancer. Crit Rev Oncol Hematol. 2011;78:79–91. 20418109 10.1016/j.critrevonc.2010.03.009

[19] Park S , Demanes J , Steinberg M , et al. PO‐289 multichannel vaginal brachytherapy: overkill or necessity? Radiother Oncol. 2012;103:S115–S116.

